# Combating Vaccine Hesitancy: Teaching the Next Generation to Navigate Through the Post Truth Era

**DOI:** 10.3389/fpubh.2018.00381

**Published:** 2019-01-14

**Authors:** Margarida Arede, Maria Bravo-Araya, Émilie Bouchard, Gurlal Singh Gill, Valerie Plajer, Adiba Shehraj, Yassir Adam Shuaib

**Affiliations:** ^1^Royal Veterinary College, University of London, Hertfordshire, United Kingdom; ^2^School of Public Health, University of Saskatchewan, Saskatoon, SK, Canada; ^3^Department of Veterinary Microbiology, University of Saskatchewan, Saskatoon, SK, Canada; ^4^Department of Veterinary Public Health and Epidemiology, Guru Angad Dev Veterinary and Animal Sciences University, Ludhiana, India; ^5^Deutsches Rheuma-Forschungszentrum, Berlin, Germany; ^6^Department of Economics, University of Saskatchewan, Saskatoon, SK, Canada; ^7^Dahlem Research School, Biomedical Science, Freie Universität Berlin, Berlin, Germany; ^8^College of Veterinary Medicine, Sudan University of Science and Technology, Khartoum, Sudan

**Keywords:** immunization, vaccine hesitancy, critical thinking, health education, children

## Abstract

Despite scientific evidence supporting the fact that vaccines are fundamental tools for preventing infectious diseases, a percentage of the population still refuses some or all of them. Vaccine hesitancy has become a widespread issue, and its complexity lies in the great variety of factors that can influence decisions about immunization, which are not just vaccine-related concerns, but also involve personal and societal levels. Our research group performed an extensive literature review to analyze: (1) different age groups, their relation to the problem and their characteristics; (2) the most important information (key messages) about immunization that could be used to counteract hesitancy; and (3) best approaches to transmit the messages to the target groups. We propose a long-term approach to overcome vaccine hesitancy that involves the education of children and adolescents on the basics about immunization and critical thinking, using different communication channels.

## Introduction

Nowadays, in the “post-truth era,” where every fact seems to be object of debate, a considerable part of the population has access to the internet and not only uses it to find information, as on health-related issues, but to create and share their own content (1, 2). This facilitates the distribution of true and false information, which canreach a large audience. Messages about vaccines on social media predominantly focus on negative experiences, since they are easier to perceive than the main benefit of vaccination: the absence of disease (3). The result is an increased disbelief of vaccine efficacy accompanied by mistrust in pharmaceutical companies (4) and subsequent rise in the incidence of vaccine hesitancy around the world (5–8).

Hesitancy to vaccinate has been linked to some vaccine preventable disease outbreaks in the last two decades. One example was the resurgence of measles in different parts of the US (9). During the year 2011, 16 measles outbreaks occurred. The effort put in place to contain these outbreaks required 42,635 to 83,133 personnel hours of work and resulted in a significant economic burden estimated to be $2.7 to $5.3 million US dollars (10). This burden was shouldered by many stakeholders including governmental health and finance departments, health insurance groups/agencies as well as NGOs and aid agencies (10). An even higher economic impact is expected in the future, with this increasing trend. Effective interventions are, therefore, urgently needed to reduce these high financial losses.

In 1999, the anti-vaxxer movement, an organized body of people who refuse to vaccinate and blaming vaccines for health problems (11, 12), got boosted when the journal Lancet published a paper claiming a correlation between the measles vaccine and autism (13). The paper was retracted 12 years later, when it was proven that several elements in it were incorrect (14); however, by this point in time, the anti-vaxxer movement had gained momentum like never before (15).

Due to the effectiveness of vaccines, health risks associated with vaccine preventable diseases are being perceived as low, which led to the cognitive bias working against the decision to vaccinate (16). Heuristics have been cursorily defined as mental shortcuts for arriving at satisfactory solutions with modest computations to allow individuals to reduce the effort associated with the decision-making process, e.g., for health risks (17). Unfortunately, heuristics often fail to produce a correct judgement, which leads to a cognitive bias, where judgement deviates from what would be considered logically desirable (18).

As vaccine hesitancy is a highly complex issue, our aim is to describe a novel approach to address it, acknowledging current efforts described in the literature and the recommendations of the World Health Organization (WHO) about this matter.

## Definition and Characteristics of the Target Group

Our policy tackles vaccine hesitancy by focusing on a novel target group: children and adolescents. We chose this audience, as most literature is addressing the current vaccine hesitancy problem in adults, in whom promoting change of attitudes toward vaccinations can be challenging. The reason for this is that the rarity of vaccine preventable diseases in developed countries has created a lack of awareness for them. Besides, parents seem to remain vaccine hesitant even after being exposed to messages designed to reduce vaccine misperception (19, 20). Although most people assume that communication about healthcare management primarily flows from parent to child, evidence exists that children can also act as behavior change agents regarding health-related issues, as health education activities brought home from school can also have a positive influence on how parents understand and manage health issues (21–24). For example, a study involving children between the age of 8 and 11, showed that teaching them about second-hand-smoke in school had a positive influence on the in-home smoking behavior of parents (23). Similarly, we expect that providing information about vaccine safety to children and adolescents in schools might lead to pro-vaccination behaviors in parents.

By targeting children and teenagers, we want to especially influence the vaccine behavior of the next generation, who will eventually become future influencers and parents themselves. As childhood and adolescence are fast pace developmental phases, different communication strategies have to be used for each age group, in order to successfully target them. Communication strategies, on which social campaign messages are based on, have evolved and it has been recognized that, to favor behavioral change, it is important to address both the individual and its surrounding (25).

In younger age groups (5 to 10 years old), the messages about health, science, and critical thinking must be kept very simple, and will function as an introduction to these topics. This would constitute a first approach before presenting more complex tasks and messages to older children and adolescents. For example, since nowadays children are exposed to online content from a very young age, giving them tools on how to verify the authenticity of the information they read could be a powerful tool to start with the development of critical thinking.

The respective characteristics and advertisement considerations for each age group, on which the recommendations of this paper were based on, are shown in Table 1.

**Table 1:** Characteristics, advertisement considerations, and preferred communication tools for children and adolescents per age group.

**Age group (Yo)**	**Characteristics**	**Advertisement considerations**	**Education at schools**	**Mass media channels**
5 to 7	- Start to become aware of health attitudes and behaviors (26) - Growing understanding of morality labels and conventional rules (26)	- More receptive to visual and audio elements (animations and voices) (26)	- Simple homework about immunization	Messages can be reinforced using television and online videos
7 to 10	- Gradually undertake peer values (27)	- Pay more attention to information they find interesting (27)	- Teaching of concepts as “herd immunity.” - Online search and internet using good practices	Information can also be endorsed on television, online videos, mobile apps, and educational shows (28)
10 to 13	- Begin to comprehend perspective and intention (29)	- Start seeking information, media use, and experimentation (29)	- Concepts about infectious diseases and eradication - Simple approaches to critical thinking are crucial.	Social media and the internet (interactive games and applications)
13 to 18	- Have more sophisticated problem-solving abilities - Reject explicit approval of adult-sponsored interests - Seeking social rewards and striving to avoid social threats (29)	- More receptive to health-related negative effects, preferring short-term effects rather than long term abstract dangers (30).	- Focus on education in critical thinking about immunization - Combination of dialogue, authentic situations, and mentorship to promote critical thinking (31)	Social media campaigns with personalized messages, focused on teenagers' interests, and motivations. Informative videos with celebrities can be spread through social media

*Yo, years old*.

## Messages to Communicate to the Target Group

### Immunization Also Protects Others

The target audience should be familiarized with the concept of herd immunity, which is defined as the proportion of immune individuals in a given population against a specific pathogen (32).

The idea that vaccines protect not just an individual, but enable us to protect others that cannot be vaccinated, could improve willingness to vaccinate (33). Highlighting the fact that there are individuals who cannot receive vaccinations e.g., against measles-mumps-rubella (MMR) as infants, immune deficient individuals, people with allergies against a vaccine's components, as well as pregnant and breastfeeding women can emphasize the role an individual's vaccination plays in a society setting (34, 35). Additionally, some people do not develop a protective immune response after vaccination and remain susceptible without knowing.

### Approved Vaccines Are Safe and Go Through Thorough Evaluations

The measles vaccine, for instance, has falsely been associated with autism, which led to lower vaccination rate coverage (36, 37). This claim is arbitrary and is not supported with sound scientific information. The risk perception for vaccination has to be improved by informing the target group in a simplified manner about vaccine development and approval, about the vaccine's influence on the frequency of outbreaks and about the probability of complications during an infection compared to side effects of the vaccine.

### Herd Immunity Can Eliminate Diseases

Partially overlapping with the first key message, this message focuses on highlighting the importance of reaching a certain immunization coverage to eradicate human pathogens as for example 95% coverage did for smallpox (38). It should also be emphasized that receiving all necessary doses of a vaccine (e.g., two doses of the MMR vaccine) are required to reach high levels of immunity.

## Communicating Policy in an Era of Misinformation

To effectively communicate the suggested public policy in the post-truth era (when emotions prevail over facts), we recommend utilizing the already existing communication tools in smart ways. Employing all types of communication channels in combination, including interpersonal, community-based, and mass media channels, is preferable as it has a better chance of changing mindsets than a single channel approach (39–41).

Utilizing the interpersonal channels of communication by teachers, doctors, and childcare personnel to communicate our key messages could be a practical solution. Teachers and doctors can communicate directly with adolescents and children to raise their awareness and sensitize them regarding specific issues like the importance of vaccination. This channel has the advantage of being the most credible source of information, highly effective, and participatory (39, 42, 43). Furthermore, it has been proven that the attitude of people toward vaccination could be changed through indirect interpersonal communication by reading about a perspective of someone who contracted a disease or by seeing pictures of diseased individuals (44).

Considering the different stages of cognitive development (Table 1) as well as culture and other factors, public health days and visuals (colorful wall graphics) could be effective community-based tools that can be utilized in schools to raise the awareness of students about the importance of vaccination, disease prevention, and other issues (45). These are credible sources of information and participatory (39, 41). Games together with simulations, using videos or GIFs, are tools to easily depict the impact of vaccination (46). Thomas et al. (47) indicated that such school-based interventions can affect students' behaviors significantly.

Mass media channels like television, radio, public transport advertising, and the internet are among the best tools to communicate public policy to all segments of a community (39). In high-income countries, adolescents spend 18 to 80 h online per week and are the predominant users of social media like Tumblr, Twitter, Instagram, Facebook, and other Apps (48, 49). Generally, the time spent by adolescents on social media is up to 4.3 h per day, while watching television and YouTube take up the second biggest portion of time (50–52). Recently, the internet became an important alternative source of health information for adolescents who avoid visiting a health professional (28). Thus, we propose that WHO, CDC, ECDC, and/or national health departments use social media platforms in order to inform the public, especially adolescents, about relevant scientific data with financial support from international and national entities.

The WHO or international health NGOs can support the suggested objective by designing videos for YouTube and TV channels to spread health information on vaccination to children and adolescents. The national offices of countries facing vaccine hesitancy would then solely need to translate the video content into the native language, providing a cost-efficient alternative. Children under the age of 12 years prefer television programs or videos with social and emotional themes, spending up to 32 h per week on screen devices (19). These videos should be visually pleasing, simple, and easy-to-follow, with a single message, and shorter than 3 min. Since social media is user-friendly and widely accessible, the videos should be shared there to reach a wider public (40). Furthermore, adolescents and children should be educated on which sources of information to trust on the internet, as mentioned in Table 1.

Communicating directly or indirectly with the secondary target groups (i.e., teachers and family), who influence perceptions, attitudes, and eventual decision making of the main target group (children and adolescents), still plays an important role. The perception of the secondary target group is mainly influenced by pediatricians, general practitioners, pediatric nurses or guidelines of schools, and daycare centers (53, 54). Therefore, continuous medical training of healthcare providers is of utmost importance to educate the secondary target group on this topic (54). However, as vaccine hesitant parents do avoid interacting with pro-vaccine-healthcare providers, they might not be reached through this approach.

It is evident that several stakeholders will be involved in facilitating the communication of our key messages. Lowering vaccine hesitancy is the most desirable for governments, the (inter)national health departments and (public) health insurances. Therefore, the finance departments at national and municipal levels are also important players. Those stakeholders will need to invest into trainings of school staff and healthcare providers to ensure effective usage of the interpersonal channel and provide funding for mass media approaches. It will thus be preferable to deliver the messages with the effort and help of the WHO SAGE, CDC, ECDC, NGOs and aid agencies, and possibly community groups, civil societies/organization, and political parties.

## Education and Critical Thinking

Critical thinking refers to purposefully reflect and reason about what to do or believe when somebody is confronted with complex issues in a specific context (55). Nowadays, many students struggle with interpreting and making reasoned decisions from a text, and with children and teenagers' increased use of social media, students should be encouraged to distinguish facts from opinions and consider relative risk (56). These skills are not intuitive, but rather learned and developed via education, and they could possibly be a long-term solution for vaccine hesitancy issues.

Unfortunately, many teachers lack instructional strategies to help adolescent students make rational decisions in a given situation, express themselves through discourse and generate their own questions (55). To achieve discussions that enhance critical analytic skills, they need to be structured and focused, but not dominated by the teacher (57). Teachers need to gradually release their control and authority to let students take more lead in discussions (56). Additionally, students need to learn how to create, evaluate and use knowledge; they need to know more than just *what*, but also *why* and *how* (31). A student who is only taught the scientific facts compared to another, who understands how science works and can build arguments, will have more difficulties to evaluate effectively different claims about controverted topics (58). The establishment of teacher professional development programs, targeting ways to promote critical thinking, would help to integrate this concept at schools (59). We suggest that this should be predominantly provided to science teachers and teachers of the country's native language and be also made available already during their studies. Even though critical thinking is best developed in an educational environment at first, this skill should be used in real-world scenarios in order to make informed decisions and engage critically in a world where information is constantly changing and shared at faster rates than ever (60). Recommendations of educational approaches to combat vaccine hesitancy, divided by age groups, are detailed in Table 1. Changes made to the educational system should preferably be implemented on a national level to avoid disparities in different regions.

## Conclusions

Vaccine hesitancy has several causes. Our approach focuses mostly on combating the spread of false information, nevertheless, we do acknowledge that emotions play an important role. Due to the drastic influence of emotions, we suggest the alternative approach of targeting children and adolescence, who might not have strong emotions about vaccines yet (and whose opinion can still be influenced through different sources). This is important, as in adults, the chances of improving risk perception solely by providing appropriate information are low due to the already established emotional connection to the topic of vaccination. Solutions should focus on communicating effectively using evidence-based information, to counteract messages that can misinform the public. In this context, and taking into consideration that critical literacy is fundamental to this matter, we propose different tools to communicate and educate children and adolescents about immunization and critical thinking, according to different developmental periods. These approaches can be applied in combination or individually, depending on the grade of vaccine hesitancy and funding available. Therefore, each country will have to define their own evaluation framework to measure the success of their particular implementation. Governments that are interested in utilizing these recommendations have to clarify first, if vaccine hesitancy is a leading cause of low vaccination rates in their country. Therefore, surveys on the vaccination status and attitude of the population should be performed using guidelines such as the one provided by the WHOs' SAGE Working Group on vaccine hesitancy (61). We acknowledge that immunization remains vulnerable to budget cuts, due to benefits not being visible immediately, but investing into prevention and health promotion, as well as communicating the importance of vaccination to young generations can have long-lasting beneficial effects in the population.

## Author Contributions

All authors listed have made a substantial, direct and intellectual contribution to the work, and approved it for publication.

### Conflict of Interest Statement

The authors declare that the research was conducted in the absence of any commercial or financial relationships that could be construed as a potential conflict of interest.
